# Prognostic Nomogram for Predicting Survival in Asian Patients With Small‐Cell Lung Cancer: A Comprehensive Population‐Based Study and External Verification

**DOI:** 10.1111/crj.70021

**Published:** 2024-11-09

**Authors:** Yuanli Xia, Jingjing Qu, Yufang Wang, Yanping Zhu, Jianying Zhou, Jianya Zhou

**Affiliations:** ^1^ The Clinical Research Center for Respiratory Diseases of Zhejiang Province, Department of Respiratory Disease, Thoracic Disease Center, The First Affiliated Hospital Zhejiang University School of Medicine Hangzhou China

**Keywords:** Asian patients, nomogram, small cell lung cancer, survival prediction

## Abstract

**Background:**

The incidence of small cell lung cancer (SCLC) among Asian patients is on the rise. Nevertheless, there remains a deficiency in precise prognostic models tailored to the specific needs of this patient population. It is imperative to develop a novel nomogram aimed at forecasting the prognosis of Asian SCLC patients.

**Methods:**

The SEER database supplied data on 661 Asian SCLC patients, who were then divided into training and internal validation sets through a random selection process. In addition, we identified 212 patients from a Chinese medical institution for the purpose of creating an external validation cohort. To forecast survival, we employed both univariate and multivariate analyses. The performance of our nomogram was assessed through calibration plots, the concordance index (C‐index), and decision curve analysis (DCA).

**Results:**

Five independent prognostic factors were determined and integrated into the nomogram. C‐index values for the training and internal validation cohorts were 0.774 (95% confidence interval [CI] = 0.751–0.797) and 0.731 (95%CI = 0.690–0.772), respectively. In the external validation cohort, the C‐index is 0.712 (95% CI = 0.655–0.7692). Calibration curves demonstrated highly accurate predictions. When compared to the AJCC staging system, our model exhibited improved net benefits in DCA. Furthermore, the risk stratification system effectively differentiated patients with varying survival risks.

**Conclusion:**

We have created a novel nomogram for predicting the survival of Asian patients with SCLC. This nomogram has been subjected to external validation and has shown its superiority over the conventional TNM staging system. It offers a more precise and reliable means of forecasting the prognosis of Asian SCLC patients.

## Graphical abstract

We have created a novel nomogram for predicting the survival of Asian patients with small cell lung cancer (SCLC). This nomogram has been subjected to external validation and has shown its superiority over the conventional TNM staging system. It offers a more precise and reliable means of forecasting the prognosis of Asian SCLC patients.

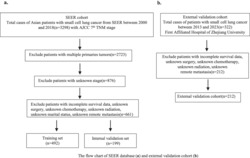

## 1 Background

Small cell lung cancer (SCLC) is a significant global public health concern, representing almost 14% of all lung cancer diagnoses, and unfortunately, the 5‐year survival rate for SCLC remains less than 7% [1], presenting global mortality to health and quality of life. SCLC is characterized by rapid proliferation, early metastases, and poor prognosis; the brain, liver, and bone are the most common metastatic sites [2]. A cohort study including 72 409 patients, the study demonstrated that the disparities in race and socioeconomic factors associated with outcomes of limited‐stage SCLC [3]. Extensive‐stage SCLC in Asian and non‐Asian patients has different survival prognoses, and the percentage of never‐smokers with SCLC appears to be higher in Asian patients; 10%–25% of patients are never‐smokers [4, 5]. Moreover, Asian and Caucasian patients with SCLC have distinct genetic characteristics, tumor microenvironments, and essential biological pathways [6].

As per the staging system established by the Veterans Administration Lung Study Group (VALSG), patients diagnosed with SCLC were typically categorized into two primary groups: extensive stage (ES‐SCLC) and limited stage (LS‐SCLC) [7]; TNM staging assigns Stages I through III to LS‐SCLC, whereas Stage IV or metastatic disease is set to ES‐SCLC [8]. The AJCC TNM staging system allows for more proper treatment selections and more precise prognostic assessments than the VALSG staging system [9]. TNM Stage I can be treated surgically or with radiation and adjuvant chemotherapy, whereas Stages I–III require concurrent chemoradiotherapy, and Stage IV chemotherapy alone or in combination with a programmed cell death protein 1 (PDL1) inhibitor is necessary [10, 11]. Nevertheless, it was clear that clinical factors, such as sex and age, were also significant predictors influencing the survival outcomes of SCLC patients, apart from TNM staging [12]. Above all, whether VALSG or TNM staging were used, they were built on most non‐Asian SCLC patients [9, 13]. Therefore, it is evident that the TNM system's limitations are insufficient for predicting the outcomes of Asian SCLC patients. Consequently, it is essential that an advanced nomogram model based on Asian SCLC patients with better prognostic discrimination is the perfect tool to increase the predictive abilities.

Nomograms has been widely used to predict the survival of SCLC patients; it is more accurate for predicting a patient's individualized prognosis than the traditional standard TNM staging [14]. The objectives of this study are to develop a prognosis nomogram of Asian patients diagnosed with SCLC and to identify critical prognostic markers.

## 2 Methods

### 2.1 SEER Cohort

**FIGURE 1:**
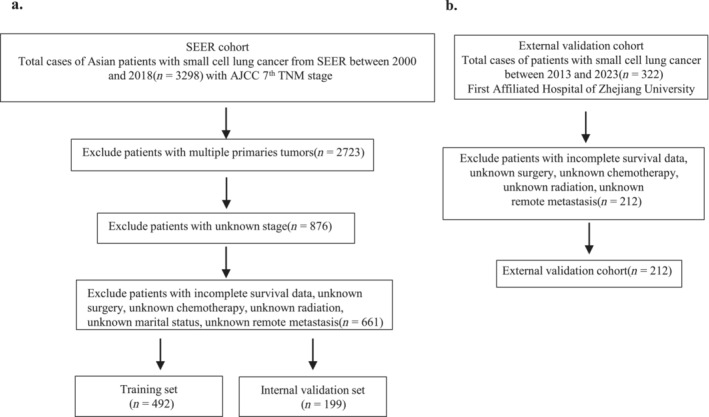
The flow chart of SEER database (a) and external validation cohort (b).

Eighteen population‐based cancer registries in the SEER Research Plus Date. By SEER*Stat software (v 8.4.1), the training and internal validation data of Asian patients with SCLC between 2000 and 2018 were collected, and 3552 Asian patients with SCLC were confirmed to meet our inclusion criteria as follows: Race/ethnicity was Chinese, Japanese, Filipino, Korean, Vietnamese, Laotian, Hmong, Kampuchean, Thai, Asian Indian, Pakistani and Other Asian; 7^th^ TNM stage, histological type of 8002/3 small cell carcinoma (ICD‐O‐3), with histological codes included as follows: small cell type, 8041/3: small cell carcinoma, NOS, 8042/3: oat cell carcinoma, 8043/3: small cell carcinoma, fusiform cell, 8044/3: small cell carcinoma, intermediate cell, and 8045/3: combined small cell carcinoma. Variables with missing values (blanks or unknown or N/A are deemed missing) and more than one primary tumor were not eligible for analysis. Age, sex, marital status, tumor location, 7^th^ TNM stage, surgery, race, chemotherapy, radiation, metastatic sites, vital status, and survival time were assessed. Eventually, after excluding the ineligible cases, 661 patients were included for analyses and those with missing data. The study's primary outcome was overall survival (OS), defined as the duration from the date of diagnosis to either the last follow‐up date or the occurrence of death. The last follow‐up in this study was conducted in November 2020 (Figure 1a).

### 2.2 External Validation Cohort

The external validation cohort was retrospectively assembled from 2013 to 2023, with the aim of evaluating the practicality of the predictive model. This cohort comprised 221 SCLC patients diagnosed at the First Affiliated Hospital, Zhejiang University School of Medicine, China. All relevant independent variables, with the exception of marital status and laterality, were collected in accordance with the SEER cohort. The last follow‐up assessment was conducted in May 2023, as depicted in Figure 1b.

### 2.3 Development and Assessment of the Prognostic Model

We conducted a Univariate COX Proportional Hazard Regression analysis to identify independent prognostic factors that could be used in the development of our prognostic model. For each independent prognostic variable, we obtained the hazard ratio (HR) along with the corresponding 95% confidence interval (CI). Factors found to be statistically significant in the univariate analysis (*p*‐value < 0.05) were then included in a multivariate COX analysis. To create the prognostic nomogram, we utilized the survival and rms packages [14].

Evaluation of a nomogram often involves looking at two different aspects: discrimination and the accuracy of the calibration [14]. The ability of a model to discriminate between patients with distinct survival outcomes is referred to as its discriminatory power [14]. The concordance index, or the C‐index, is usually used to quantify discrimination [14]. Under the receiver operating characteristic (ROC) curve, the C‐index is a measure of concordance comparable to the area [14]. The calibration accuracy is measured by looking at how well the projected probability matches the actual survival results, which are displayed as calibration curves [14]. Utilizing R's survival and rms package allowed for the generation of calibration curves for the nomogram's OS estimates [14].

To conduct a more thorough assessment of the benefits and advantages offered by our newly developed predictive model, we utilized decision curve analysis (DCA). DCA, commonly utilized, compares alternative diagnostic and prediction methodologies with other widely employed metrics and methods, all of which come with their own set of distinctive benefits.

### 2.4 Building a Risk Stratification

The patients in our cohort were classified into two distinct risk categories, namely, low risk and high risk, based on the cumulative score obtained from individual assessments using the nomogram. We determined the most suitable cutoff value for our purposes through X‐title analysis. To assess the efficacy of our risk stratification model, we conducted Kaplan–Meier survival analyses in both the training and external validation cohorts. Additionally, we used log‐rank tests to evaluate differences in survival outcomes among various risk groups. We considered *p*‐values less than 0.05 (two‐sided) as indicative of statistical significance. All statistical analyses were performed using R version 4.3.0 software.

## 3 Results

### 3.1 Characteristics of the SEER and External Validation Cohort

Finally, following the stepwise selection, a total of 661 cases from the SEER database were included in the study. These patients were randomly allocated into two cohorts, with a split ratio of 7:3. The training cohort consisted of 462 patients, while the internal validation cohort comprised 199 patients. Table 1 provides a summary of the clinical features of the patients. In the SEER cohort, 16.8% of the cases were Chinese, 18.6% of the cases were Japanese, and 64.6% were other Asian, including Korean, Thai, Vietnamese, Indian, Filipino, Hmong, Kampuchean, Laotian, Pakistani, and other Asian. A greater percentage of males (70.5%) were observed compared to females (29.5%), with over half of the patients being in Stage IV. All clinicopathological characteristics were similar between the training and internal validation cohorts. In the external validation cohort, 93 patients (43.9%) fell within the age range of 65 to 75 years, 183 (86.3%) patients were male, and 15 (7.1%), 208 (98.1%), and a total of 132 (62.3%) received surgery, chemotherapy, and radiation, respectively. A total of 131 (61.8%) patients were Stage IV with distant metastasis.

**TABLE 1:** Patient characteristics.

Variables	Total (*N* = 661)	Training cohort (*N* = 462)	Validation cohort (*N* = 199)	*p*‐value	External validation(*N* = 212)	*p*‐value
Age (year) *n* (%)				0.924		0.000
<65	183 (27.7%)	134 (29.0%)	49 (24.6%)		86 (40.6%)	
65–75	239 (36.2%)	159 (34.4%)	80 (40.2%)		93 (43.9%)	
>75	239 (36.2%)	169 (36.6%)	70 (35.2%)		33 (15.6%)	
Sex *n* (%)				0.682		0.000
Female	195 (29.5%)	139 (30.1%)	56 (28.1%)		29 (13.7%)	
Male	466 (70.5%)	323 (69.9%)	143 (71.9%)		183 (86.3%)	
Race/ethnicity *n* (%)				0.639		—
Chinese	111 (16.8%)	76 (16.5%)	35 (17.6%)			
Japanese	123 (18.6%)	88 (19.0%)	35 (17.6%)			
Other Asian	427 (64.6%)	298 (64.5%)	129 (64.8%)			
AJCC TNM stage (7^th^) *n* (%)				0.987		0.279
I	24 (3.6%)	16 (3.5%)	8 (4.0%)		2 (0.9%)	
II	29 (4.4%)	19 (4.1%)	10 (5.0%)		8 (3.8%)	
III	183 (27.7%)	142 (30.7%)	41 (20.6%)		71 (33.5%)	
IV	425 (64.3%)	285 (61.7%)	140 (70.4%)		131 (61.8%)	
Surgery *n* (%)				0.186		0.001
No	647 (97.9%)	454 (98.3%)	193 (97.0%)		197 (92.9%)	
Yes	14 (2.1%)	8 (1.7%)	6 (3.0%)		15 (7.1%)	
Chemotherapy *n* (%)				0.349		0.000
No	187 (28.3%)	132 (28.6%)	55 (27.6%)		4 (1.9%)	
Yes	474 (71.7%)	330 (71.4%)	144 (72.4%)		208 (98.1%)	
Radiation *n* (%)				0.004		0.027
No	315 (47.7%)	218 (47.2%)	97 (48.7%)		80 (37.7%)	
Yes	346 (52.3%)	244 (52.8%)	102 (51.3%)		132 (62.3%)	
Marital status *n* (%)				0.462		—
Unmarried/single	56 (8.5%)	39 (8.4%)	17 (8.5%)			
Married	605 (91.5%)	423 (91.6%)	182 (91.5%)			
Laterality *n* (%)				0.897		—
Left	259 (39.2%)	180 (39.0%)	79 (39.7%)			
Right	376 (56.9%)	266 (57.6%)	110 (55.3%)			
Other	26 (3.9%)	16 (3.5%)	10 (5.0%)			
Bone. metastasis *n* (%)				0.239		0.009
No	537 (81.2%)	382 (82.7%)	155 (77.9%)		156 (73.6%)	
Yes	124 (18.8%)	80 (17.3%)	44 (22.1%)		56 (26.4%)	
Brain. metastasis *n* (%)				0.224		0.06
No	553 (83.7%)	383 (82.9%)	170 (85.4%)		162 (76.4%)	
Yes	108 (16.3%)	79 (17.1%)	29 (14.6%)		50 (23.6%)	
Liver. metastasis *n* (%)				0.087		0.364
No	520 (78.7%)	373 (80.7%)	147 (73.9%)		164 (77.4%)	
Yes	141 (21.3%)	89 (19.3%)	52 (26.1%)		48 (22.6%)	

### 3.2 Risk Factors for OS

Univariate COX regression analyses revealed significant associations between OS and factors such as age, AJCC TNM stage, chemotherapy, radiation, and the presence of liver metastasis (Table 2). However, sex, race/ethnicity, surgery, marital status, laterality, bone metastasis, and brain metastasis did not show significance for OS. All five significant factors, age (*p* < 0.001), AJCC TNM stage (*p* < 0.001), chemotherapy (*p* < 0.001), radiation (*p* < 0.001), liver metastasis(*p* = 0.005), the factors eventually included in the multivariate COX regression analysis, were found to be standalone prognostic indicators (Table 2).

**TABLE 2:** Cox regression analyses of prognostic variables for OS.

	Univariate analysis		Multivariate analysis	
Variables	HR(95% CI)	*p*‐value	HR(95% CI)	*p*‐value
Age (year) *n* (%)				
<65	Reference			
65–75	1.10 (0.89–1.36)	0.371	Reference	
>75	1.50 (1.21–1.87)	<0.001	1.55 (1.25–1.93)	<0.001
Sex *n* (%)			1.11 (0.90–1.37)	0.329
Male	Reference			
Female	0.84(0.70–1.01	0.066		
Race/ethnicity *n* (%)				
Chinese	Reference			
Japanese	1.27 (0.96–1.67)	0.093		
Other Asian	1.06(0.85–1.33)	0.585		
AJCC TNM stage (7^th^) *n* (%)				
I	Reference		Reference	
II	1.79 (0.97–3.29)	0.062	1.79 (0.99–3.25)	0.056
III	2.47 (1.52–4.02)	<0.001	2.47 (1.54–3.97)	<0.001
IV	4.41 (2.70–7.21)	<0.001	4.30 (2.70–6.85)	<0.001
Surgery *n* (%)				
No	Reference			
Yes	0.84 (0.45–1.57)	0.582		
Chemotherapy *n* (%)				
No	Reference		Reference	
Yes	0.38 (0.31–0.46)	<0.001	0.39 (0.32–0.47)	<0.001
Radiation *n* (%)				
No	Reference	<0.001	Reference	
Yes	0.61 (0.51–0.74)		0.60 (0.51–0.72)	<0.001
Marital status *n* (%)				
Unmarried/Single	Reference			
Married	1.10 (0.81–1.49)	0.558		
Laterality *n* (%)				
Left	Reference			
Right	0.99 (0.83–1.17)	0.901		
Other	0.99 (0.64–1.52)	0.959		
Bone metastasis *n* (%)				
No	Reference			
Yes	1.10(0.88–1.37)	0.426		
Brain metastasis *n* (%)				
No	Reference			
Yes	0.91(0.71–1.16)	0.428		
Liver metastasis *n* (%)				
No	Reference		Reference	
Yes	1.28 (1.03–1.59)	0.029	1.35 (1.10–1.67)	0.005

Abbreviations: CI, confidence interval; HR, hazard ratio; OS overall survival.

### 3.3 Establishment and Verification of Nomogram

**FIGURE 2:**
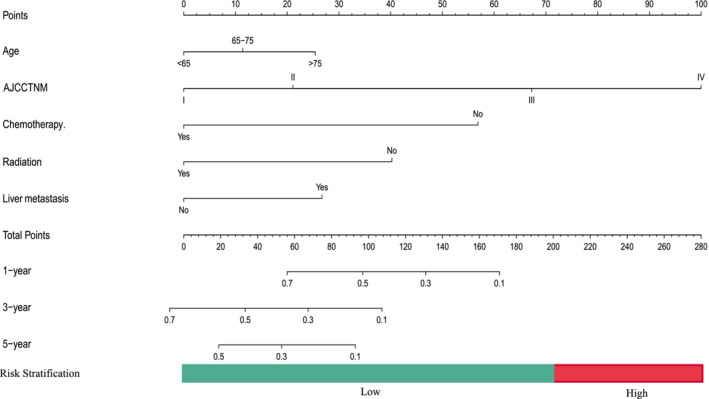
Nomogram predicting 1‐, 3, and 5‐year OS of Asian patients with small cell lung cancer.

**FIGURE 3:**
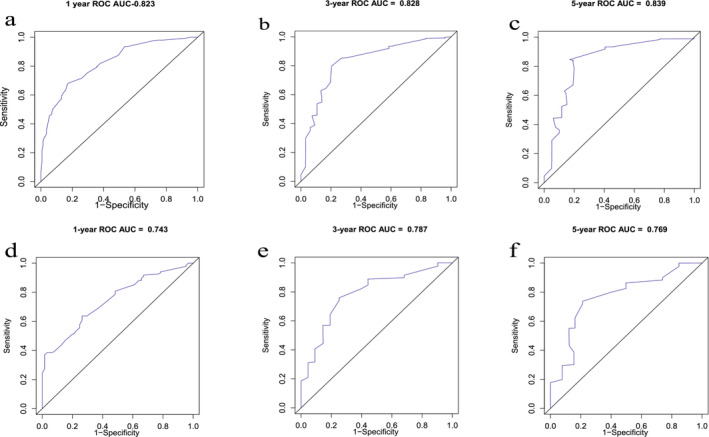
Nomogram by Receiver operating characteristic (ROC) analyses. (a–c) ROC curves for discrimination in the training cohort for 1‐year (a), 3‐year (b), and 5‐year (c) overall survival. (d–f) ROC curves for discrimination in the internal validation cohort for 1‐year (d), 3‐year (e), and 5‐year (f) overall survival. AUC area under the curve.

A nomogram was built based on five prognostic indicators that were significantly associated with OS, age, AJCC TNM stage, chemotherapy, radiation, and liver metastasis for 1‐, 3‐ and 5‐year OS (Figure 2). The C‐index for our new nomogram in the training cohort was 0.774 (95% CI = 0.751–0.797), while the C‐index for clinical staging was 0.661 (95% CI = 0.609–0.713), with a statistically significant difference between the two (*p* < 0.001). In the internal validation cohort, the C‐index for our new nomogram reached 0.731 (95% CI = 0.690–0.772), while the C‐index for clinical staging reached 0.603 (95% CI = 0.532–0.675), with a statistically significant difference between the two (*p* = 0.023), as shown in (Figure 3). The performance of the nomogram is showcased by the area under the curve (AUC) values, specifically at 1‐year, 3‐year, and 5‐year OS, both in the training group (1‐year: 0.823, 3‐year: 0.828, and 5‐year: 0.839) and the internal validation group (1‐year: 0.743, 3‐year: 0.787, and 5‐year: 0.769). These AUC values attest to the nomogram's exceptional discrimination ability.

**FIGURE 4:**
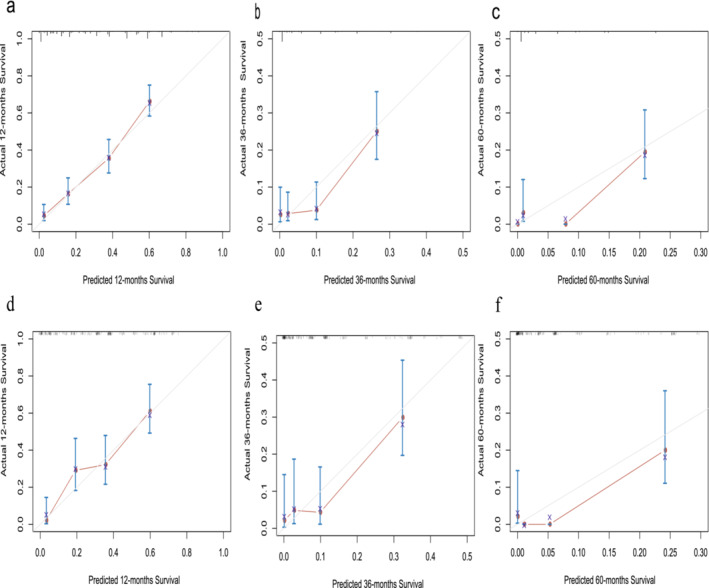
Calibration curves. (a–c) The calibration curves of the model for (a) 1‐year, (b) 3‐year, and 5‐year and (c) overall survival in the training cohort. (d–f) The calibration curves of the model for (d) 1‐year, (e) 3‐year, and 5‐year and (f) overall survival in the validation cohort. *Y*‐axis indicated the actual survival probability and *x*‐axis indicated the predicated survival probability. The gray line indicated that prediction agrees with actuality. Error bars represent 95% confidence intervals.

Furthermore, when examining the calibration plots for 1‐year, 3‐year, and 5‐year OS probabilities, they reveal a high degree of agreement between the nomogram's predicted survival and the actual observations. This consistency was observed in both the training and internal validation cohorts (Figure 4).

### 3.4 Nomogram and 7^th^ TNM Staging System Comparison

**FIGURE 5:**
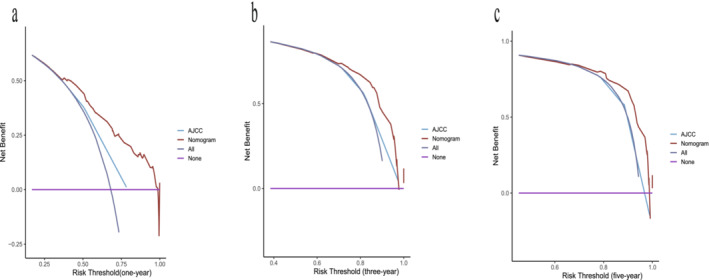
Decision curve analyses (DCA) of the nomogram and 7^th^ edition AJCC TNM staging system for 1‐year (a), 3‐year (b), and 5‐year and (c) overall survival. The *x*‐axis represents the threshold probabilities, and the *y*‐axis measures the net benefit. The horizontal line along the *x*‐axis assumes that overall death occurred in no patients, whereas the solid gray line assumes that all patients will have overall death at a specific threshold probability.

The findings from the DCA investigations clearly indicate that our newly developed nomogram provides significant net benefits when compared to the 7^th^ AJCC TNM staging system. This advantage is noticeable across a wide and practical range of threshold probabilities in both the training and internal validation cohorts, as illustrated in (Figure 5). These results underscore the enhanced utility of the updated nomogram in clinical settings, particularly for predicting individual survival outcomes.

### 3.5 External Validation of a Nomogram

**FIGURE 6:**
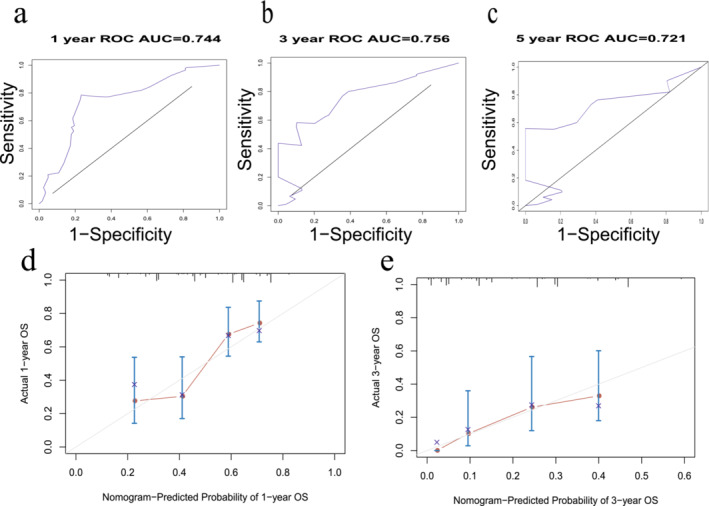
Nomogram by receiver operating characteristic (ROC) analyses. (a–c) ROC curves for discrimination in the external validation cohort for 1‐year (a), 3‐year (b), and 5‐year (c) overall survival. Calibration curves, the calibration curves of the model for (d) 1‐year, (e) 3‐year, overall survival in the external validation cohort.

In terms of the AUC for OS prediction at 1, 3, and 5 years, we obtained values of 0.744, 0.756, and 0.721, as illustrated in Figure 6a–c, respectively. Furthermore, when examining the calibration curves (as shown in Figure 6d,e), we observed excellent agreement between the predicted survival probabilities and the actual observed outcomes for 1‐ and 3‐year survival.

### 3.6 Performance of the new Risk Stratification Model

**FIGURE 7:**
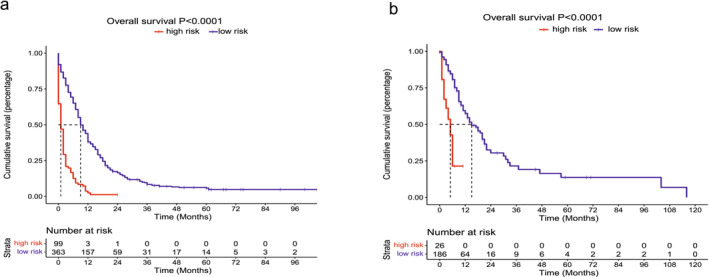
Kaplan–Meier survival analyses to test the risk stratification system within the training (a) and the external validation cohort (b).

Using the nomogram model, we established a risk categorization for patients based on their cumulative scores within the training cohort. To distinguish between high‐risk and low‐risk groups, we applied a threshold value of 192.38. Subsequently, all patients were categorized into either the high‐risk group (total points > 192.38) or the low‐risk group (total points ≤ 192.38). Within the training cohort, our analysis unveiled a substantial discrepancy in OS between high‐risk and low‐risk patients. High‐risk patients experienced significantly worse OS outcomes when compared to their low‐risk counterparts (*p* < 0.0001), as evidenced by Kaplan–Meier survival analyses (Figure 7a). Additionally, with the cutoff point of the established nomogram, two risk stratifications were divided to evaluate OS. In the external validation cohort, our analysis indicated that the high‐risk cohort experienced markedly inferior OS compared to the low‐risk cohort, with a statistically significant difference (*p* < 0.0001) (Figure 7b). This result is consistent with the training cohort.

### 3.7 User Friendly Tool

Utilizing the newly developed nomogram, we created a user‐friendly tool for predicting the survival of Asian SCLC patients. This tool is accessible at https://sinbad.shinyapps.io/DynNomapp/ and is designed to be easily used by healthcare professionals, eliminating the need for a password.

## 4 Discussion

To our knowledge, there is no specific nomogram model to predict the prognosis of the Asian SCLC population. This is the first nomogram model in which the patients in the training and external validation cohorts were all Asian SCLC patients since the previous studies were based on the SEER all‐race population of database analysis and whether the model can be applied to the Asian population remains uncertain [15]. Although SCLC is generally known as an aggressive malignancy, the VALSG or TNM staging approach is poor in predicting the individualized OS of SCLC patients. Consequently, we developed and subjected to external evaluation a clinical prognostic model for Asian SCLC, which provides survival estimates based on clinical factors. The newly developed model exhibited great discriminating capacity as well as calibration accuracy. Our innovative nomogram included five variables: age, AJCC TNM stage, chemotherapy, radiation, and liver metastasis. Compared to the TNM staging approach, the newly developed model produced a more accurate prognosis for Asian SCLC patients.

Certain studies provide useful nomograms for doctors to classify the risk of SCLC patients. Nevertheless, these studies had some limitations: Pan et al. established a novel nomogram for SCLC patients, 275 Chinese patients were included in a single center cohort as the training set, and only 80 patients were further validated in an independent cohort [16]. Wang et al. developed a nomogram model for SCLC patients using the National Cancer Database (NCDB) of the US; 98% of patients were white and black, although the author used an internal validation cohort, which limits its generalizability [17]. Xie et al. used the Mayo Clinic Tumor Registry and Social Security Death Index website to build a nomogram for SCLC [18]. Li et al. and Xiao et al. used an internal validation of the training dataset [19, 20], In contrast, we first included Asian patients in the SEER and external validation SCLC cohorts to obtain a better understanding of SCLC.

We extracted the Asian cohort from SEER; in our Asian cohort, 16.8% of cases were Chinese, 18.6% of patients were Japanese, and 64.6% were other Asian, including Korean, Thai, Vietnamese, Indian, Filipino, Hmong, Kampuchean, Laotian, Pakistani, and others. Additionally, we found five independent predictive variables for OS, age, AJCC TNM stage, chemotherapy, radiation, and liver metastasis, consistent with previous findings. Interestingly, our study also indicated that sex, brain metastases, and surgery are not prognostic factors for Asian SCLC patients, and these results were not consistent with other non‐Asian SCLC studies [21, 22, 23]. There is a relatively small body of literature that is concerned with the differences between Asian and non‐Asian SCLC; a key study showed the differences between Asian and Caucasian individuals regarding genomic characteristics, tumor microenvironment, and critical biological pathways [6]. This study enhanced our knowledge of racial differences among Asian and Non Asian SCLC patients.

Chemotherapy with etoposide and platinum (EP) has remained the standard first‐line treatment in extensive‐SCLC; median survival with this treatment is only 10 months. Since 46.3% of the patients in the study had stage IV SCLC, the majority of patients in the training group had a survival period of less than 2 years. As a result, it is expected that the calibration curves and ROC curves at the 3‐year and 5‐year time points would show some deviations. This is because, over time, the number of surviving patients significantly decreases, leading to reduced predictive accuracy of the model at these longer time points compared to the short‐term predictions. This deviation reflects the limitations of the model under conditions of data sparsity, particularly in long‐term predictions. Therefore, while the model performs well in short‐term predictions, it is important to consider this uncertainty when interpreting and utilizing the 3‐year and 5‐year prediction results. In the future, during model development and optimization, using a larger dataset could help improve the model's performance at these time points.

External validation of prediction models is crucial for establishing their generalizability and preventing overfitting [24]. It was confirmed by calibration plots that the developed nomograms were repeatable and reliable in the external validation cohort when compared to actual observed 1‐, 3‐, and 5‐year OS and the predictions made by the nomogram. Moreover, the validation cohort displayed higher C‐index and AUC values for our model compared to a previously established SCLC nomogram [16]. Furthermore, the external validation cohort of patients could be effectively stratified into high‐risk and low‐risk groups. Our study also suggests that the following features of a high‐risk SCLC patient are present: age >75, Stage IV, liver metastases, lack of radiation treatment, and chemotherapy.

This study has limitations that need to be noted. Initially, it is important to note that the training and external validation datasets in this study were obtained through retrospective collection, potentially introducing inherent biases. Second, it is worth noting that certain factors related to treatment, including the particular chemotherapy and immunotherapy drugs, as well as distinct disease characteristics such as comorbidities, body mass index (BMI), smoking habits, and dietary patterns, all of which could have an impact on prognosis, were not incorporated into this study. Third, external cohorts were collected from a single institution, and the sample size was not large. Finally, the AJCC 8^th^ staging system for Asian SCLC patients from the SEER database is few; it is not suitable for analysis, so we collected the AJCC 7^th^ staging system for analysis. In spite of these limitations, our nomogram, tailored specifically for Asian SCLC patients using data from the SEER database and external validation, holds significant promise in accurately predicting OS for this patient population. Looking forward, we plan to expand and enhance the model's predictive capabilities by incorporating additional factors from diverse databases.

## 5 Conclusion

We developed both a nomogram and an associated risk classification system for forecasting OS in Asian SCLC patients. The model exhibited strong performance when validated with an external cohort. We offer a user‐friendly tool that facilitates swift calculations in clinical settings, aiding Asian medical professionals in making informed clinical decisions and patient stratification. Simultaneously, this model may serve as a foundation for researchers seeking to define appropriate stratification parameters in future clinical trials. It is important to note that additional studies are warranted to validate the prognostic utility of this model.

## Author Contributions

Y.L. drafted the manuscript, designed, and conducted statistics analyses; J.J. collected the external data. Y.F. and Y.P. drew the figures and tables. J.Y. and J.Y. contributed to revision. All authors read and approved the final manuscript.

## Ethics Statement

The data of this study are obtained from the SEER database. The patients' data are public and anonymous, so this study does not require ethical approval and informed consent, and the Institutional Review Board of the First Affiliated Hospital, Zhejiang University School of Medicine, approved the external validation cohort.

## Consent

All authors approve this manuscript for publication.

## Conflicts of Interest

The authors declare no conflicts of interest.

## Data Availability

The data analyzed in this study are available online (https://seer.cancer.gov/).
